# Supplementary material for: Unlicensed Molnupiravir is an Effective Rescue Treatment Following Failure of Unlicensed GS-441524-like Therapy for Cats with Suspected Feline Infectious Peritonitis
Source: Pathogens. 2022 Oct 20;11(10):1209. doi: 10.3390/pathogens11101209 (PMC9612227; doi:10.3390/pathogens11101209)
Supplement: Supplementary file 1 [file pathogens-11-01209-s001.zip › pathogens-1952332-supplementary.pdf]

Data S1:Molnupiravir Retrospective Study

Start of Block: Consent

Q60
The Ohio State University Consent to Participate in Research

Study Title: Is Unlicensed Molnupiravir Effective for Treatment of Feline Infectious Peritonitis following Failure of Unlicensed GS-441524-Like Therapy?

Protocol Number: 2021E0162

Researcher: Samantha J.M. Evans, DVM, PhD, DACVP

Sponsor: OSU Veterinary Summer Research Program

This is a consent form for research participation. It contains important information about this study and what to expect if you decide to participate. It contains important information about this study and what to expect if you decide to participate.

Your participation is voluntary.

Please consider the information carefully. Feel free to ask questions before making your decision whether or not to participate.

Purpose: We are researching the use of unlicensed antiviral therapeutic compounds for treatment of feline infectious peritonitis (FIP).

Procedures/Tasks: Cat owners who have used unlicensed drugs to treat their cats will take a single online survey.

Duration: The surveys will take approximately 20-25 minutes to complete.

You may leave the study at any time. If you decide to stop participating in the study, there will be no penalty to you, and you will not lose any benefits to which you are otherwise entitled. Your decision will not affect your future relationship with The Ohio State University.

Risks and Benefits: There are no direct benefits to the participants, other than helping us to learn more about what factors determine success for future cats. There are no risks to participants.

Confidentiality:

We will work to make sure that no one sees your online responses without approval. But, because we are using the Internet, there is a chance that someone could access your online responses without permission. In some cases, this information could be used to identify you.

Also, there may be circumstances where this information must be released. For example, personal information regarding your participation in this study may be disclosed if required by state law. Also, your records may be reviewed by the following groups (as applicable to the research): Office for Human Research Protections or other federal, state, or international regulatory agencies The Ohio State University Institutional Review Board or Office of Responsible Research Practices

No such breach of confidentiality is expected.

Future Research: Your de-identified information may be used or shared with other researchers without your additional informed consent.

Incentives: There are no financial incentives for participating in this research.

Participant Rights:

You may refuse to participate in this study without penalty or loss of benefits to which you are otherwise entitled. If you are a student or employee at Ohio State, your decision will not affect your grades or employment status.

If you choose to participate in the study, you may discontinue participation at any time without penalty or loss of benefits. By agreeing to participate, you do not give up any personal legal rights you may have as a participant in this study.
This study has been determined Exempt from IRB review.

Contacts and Questions:

For questions, concerns, or complaints about the study you may contact Dr. Samantha Evans at evans.2608@osu.edu .

For questions about your rights as a participant in this study or to discuss other study-related

concerns or complaints with someone who is not part of the research team, you may contact the Office of Responsible Research Practices at 1-800-678-6251 or hsconcerns@osu.edu.

Providing consent

I have read (or someone has read to me) this page and I am aware that I am being asked to participate in a research study. I have had the opportunity to ask questions and have had them answered to my satisfaction. I voluntarily agree to participate in this study. I am not giving up any legal rights by agreeing to participate.

To print or save a copy of this page, select the print button on your web browser.

Please select "I agree" below to proceed and participate in this study. If you do not wish to participate, please select "I disagree" or close out your browser window. “

○ I agree

○ I disagree

End of Block: Consent

Start of Block: General Information About Your Cat

Q53 General Information About You and Your Cat
What is your email address?

____

Q61 In which country do you currently reside?

▼ Afghanistan ... Zimbabwe

Q1 What is your cat’s name?

____

Q3 What was the sex/neuter status of your cat at the time of their FIP diagnosis?

- ○ Female (not spayed)
- ○ Male (not neutered)
- ○ Spayed Female
- ○ Neutered Male

Q7 What is your cat's breed? (Please check all that apply)

- ☐ Domestic Mixed-Breed/"Moggie"
- ☐ Abyssinian
- ☐ American Bobtail
- ☐ American Curl
- ☐ American Shorthair
- ☐ American Wirehair
- ☐ Balinese
- ☐ Bengal
- ☐ Birman
- ☐ Bombay
- ☐ British Shorthair
- ☐ Burmese
- ☐ Burmilla
- ☐ Chartreux
- ☐ Chausie
- ☐ Colorpoint Shorthair
- ☐ Cornish Rex
- ☐ Devon Rex

- [ ] Egyptian Mau
- [ ] European Burmese
- [ ] Exotic
- [ ] Havana Brown
- [ ] Himalayan
- [ ] Japanses Bobtail
- [ ] Khao Manee
- [ ] Korat
- [ ] LaPerm
- [ ] Lykoi
- [ ] Maine Coon
- [ ] Manx
- [ ] Norwegian Forest Cat
- [ ] Ocicat
- [ ] Oriental
- [ ] Persian (including Himalayan)
- [ ] RagaMuffin
- [ ] Ragdoll

- [ ] Russian Blue
- [ ] Savannah
- [ ] Sottish Fold
- [ ] Selkirk Rex
- [ ] Serengeti
- [ ] Siamese
- [ ] Siberian
- [ ] Singapura
- [ ] Somali
- [ ] Sphynx
- [ ] Tonkinese
- [ ] Toybob
- [ ] Toyger
- [ ] Turkish Angora
- [ ] Turkish Van
- [ ] Other: (please specify)

____

- [ ] Unknown/not sure

JS

Q5 What is your cat's birthdate? (If you don't know the exact date, your best guess is fine.)

Month	▼ January ...
Day	▼ January ...
Year	▼ January ...

JS

Q68 What date was your cat diagnosed with FIP? (If you don't know the exact date, your best guess is fine.)

Month	▼ January ...
Day	▼ January ...
Year	▼ January ...

Q15 Did your cat have any pre-existing medical conditions before they were diagnosed with FIP?

◯ Yes

◯ No

Q23 Please select your cat's existing medical conditions from the following list. (Select all that apply)

- ☐ Acute kidney injury (AKI)
- ☐ Asthma
- ☐ Bladder stones/crystals
- ☐ Calicivirus
- ☐ Cancer: (please specify type) ____
- ☐ Cerebellar Hypoplasia
- ☐ Chronic kidney disease (CDK)
- ☐ Conjunctivitis
- ☐ Dental disease (eg. gingivitis, stomatitis, resorptive lesions): (please specify) ____
- ☐ Diabetes
- ☐ Epilepsy
- ☐ Feline herpes virus (FHV-1)
- ☐ Feline idiopathic cystitis (FIC), aka feline lower urinary tract disease (FLUTD)
- ☐ Hepatic lipidosis
- ☐ Hyperthyroidism
- ☐ Hypertrophic cardiomyopathy (HCM)

- [ ] Immune-mediated hemolytic anemia (IMHA)
- [ ] Inflammatory bowel disease (IBD)
- [ ] Megacolon
- [ ] Mycobacterium infection
- [ ] Mycoplasma-related anemia
- [ ] Panleukopenia
- [ ] Parasites (eg. intestinal worms, giardia, coccidia, heartworm, fleas): (please specify) ____
- [ ] Ringworm
- [ ] Skin disease (eg. ringworm, bacterial infection, ear infection, atopic/allergic dermatitis) : (please specify) ____
- [ ] Toxoplasmosis
- [ ] Upper respiratory infection (URI) of unknown cause
- [ ] Urinary or fecal incontinence (non-FIP related)
- [ ] Urinary tract infection (UTI)
- [ ] Other: (please specify) ____

End of Block: General Information About Your Cat

Start of Block: FIP Diagnosis

Q8 Was your cat diagnosed with FIP by a veterinarian?

- ◯ Yes
- ◯ No

Q7 FIP Diagnosis and Symptoms

Which of the following diagnostics were performed by your vet to help diagnose FIP? (Please check all that apply)

- [ ] Abdominal ultrasound
- [ ] Biopsy with IHC (immunohistochemical staining)
- [ ] Biopsy without IHC (immunohistochemical staining) - histopathology only
- [ ] CBC (complete blood count; a common type of blood test)
- [ ] Chemistry panel (AKA biochemical profile; a common type of blood test)
- [ ] CT (AKA "cat scan")
- [ ] Cytology (via fine needle aspirate)
- [ ] ECG/EKG (electrocardiogram)
- [ ] Feline coronavirus (FCoV) RT-PCR or titer
- [ ] FIP ELISA 7b protein test (by Antech Laboratories)
- [ ] FIP mRNA PCR or IFA (immunofluorscence assay)
- [ ] FIP Virus RealPCR test (by IDEXX Laboratories)
- [ ] Fluid analysis: cerebrospinal fluid (CSF) from a spinal tap
- [ ] Fluid analysis: complete fluid analysis of effusion fluid
- [ ] Fundic exam (a specific type of eye exam)
- [ ] Neurological exam

- [ ] MRI
- [ ] Radiographs (x-rays)
- [ ] Rivalta test (test conducted on effusion fluid)
- [ ] Other: (please specify)

____

- [ ] ⊗ None - no specific diagnostic tests were performed by my veterinarian

---

Q8 Do you have results for any of these diagnostic tests?

- ○ Yes (Please upload them at the end of the survey, if possible!)
- ○ No

Q62 What type(s) of FIP was your cat diagnosed with? (Please check all that apply)

- [ ] Effusive (wet)
- [ ] Non-effusive (dry)
- [ ] Neurological
- [ ] Ocular
- [ ] ⊗ Unknown/Not Sure

Q10 How was a diagnosis of FIP achieved?

____

Q14 Did YOU observe your cat experiencing any of the following symptoms around the time of diagnosis? (Please check all that apply)

- ☐ Anisocoria (different sized-pupils)
- ☐ Blindness
- ☐ Color changes or spots in the eye
- ☐ Constipation/obstipation (difficulty defecating)
- ☐ Diarrhea
- ☐ Difficulty breathing
- ☐ Difficulty walking or jumping
- ☐ Distended abdomen (swollen belly due to fluid build-up)
- ☐ Hiding/lack of socialization
- ☐ Incontinence: fecal (loss of ability to control bowel movements)
- ☐ Incontinence: urinary (loss of bladder control)
- ☐ Increased water consumption
- ☐ Jaundice (yellowed skin or eyes)
- ☐ Lethargy (low energy)
- ☐ Loss of appetite
- ☐ Pale gums
- ☐ Paralysis (partial or total)

- [ ] Pica (eating/licking of inappropriate objects, eg. platic bags, paper, kitty litter)
- [ ] Seizures (either focal or grand mal)
- [ ] Tremors or shaking
- [ ] Upper respiratory issues (ie. sneezing, congestion, nasal discharge)
- [ ] Vomiting
- [ ] Weight loss
- [ ] Other: (please specify)

- [ ] ⊗ No symptoms/issues were observed

Q37 Around the time of your cat's FIP diagnosis, was your cat diagnosed as having any of the following conditions by a veterinarian? (Please check all that apply)

- ☐ Anisocoria (pupils are different sizes)
- ☐ Blindness
- ☐ Cardiomegaly (enlarged heart)
- ☐ Enlarged lymph nodes
- ☐ Granulomas: intestinal (inflammatory nodules in the intestines)
- ☐ Granulomas: lung (inflammatory nodules in the lungs)
- ☐ Heart murmur
- ☐ Hepatic injury or insufficiency (liver disease or failure)
- ☐ Hepatomegaly (enlarged liver)
- ☐ Hypertrophic cardiomyopathy (HCM) (a form of heart disease)
- ☐ Kidney disease
- ☐ Nephromegaly (enlarged kidney(s))
- ☐ Pulmonary edema (excess fluid within the lungs)
- ☐ Retinal detachment (detached layer at the back of the eye, causing blindness)
- ☐ Upper respiratory infection (URI)
- ☐ Uveitis (a form of eye inflammation)
- ☐ Other: (please specify)

____

☐ ⊗ No other medical conditions were diagnosed by my veterinarian

End of Block: FIP Diagnosis

Start of Block: First Treatment of FIP

Q11 What was the initial therapy used to treat your cat for FIP?

○ Oral GS-441524

○ Injectable GS-441524

○ Combination of oral and injectable GS-441524

○ Combination of GS-441524 and GC

○ GC376

○ Remdesivir

○ Molnupiravir

○ Other (please specify) ____

Q13 GS-441524 Therapy Questions

What dose of GS-441524 therapy did you first use for your cat?

○ 4-5 mg/kg

○ 5-6 mg/kg

○ 6-7 mg/kg

○ 7-8 mg/kg

○ Other, please explain ____

Q76 How often did you administer GS-441524 therapy to your cat?

- ○ Once a day
- ○ Twice a day
- ○ Other, please explain ____

Q69 Did the dose of GS-441524 therapy change at any point during the course of treatment?

- ○ Yes
- ○ No

Q70 Please explain how the dosage of GS-441524 therapy changed during the course of treatment.

____

____

____

____

____

Q56 What dose and frequency of GC therapy did you use for your cat? Please indicate if this dose changed during the course of therapy.

____

Q75 What dose of GS-441524 and what dose of GC did you use to treat your cat?

- ○ Dose of GS-441524 ____
- ○ Dose of GC ____

Q77 How often did you administer GS-441524 and GC to your cat?

- ○ Both therapies once a day
- ○ Both therapies twice a day
- ○ Other, please explain ____

Q57 What dose and frequency of remdesivir therapy did you use for your cat? Please indicate if this dose changed during the course of therapy.

____

Q78 What dose and frequency of molnupiravir therapy did you use for your cat? Please indicate if this dose changed during the course of therapy.

____

Q58 What dose and frequency of this therapy did you use for your cat? Please indicate if this dose changed during the course of therapy.

____

JS

Q25 Initial Therapy Section

What date did you start the initial therapy for FIP? (ex: GS, GC, remdesivir)

Month	▼ January ...
Day	▼ January ...
Year	▼ January ...

Q12 Did your pet finish the standard 12 week (84 days) course of the initial therapy?

◯ Yes

◯ No (Please explain) ____

Q24 Did you extend the course of the initial therapy past 12 weeks (84 days)?

◯ Yes (Please include how long you extended treatment for in days)
____

◯ No

Q16 Did you miss a dose of the initial therapy for any reason?

◯ Yes (Please explain) ____

◯ No

JS

Q26 What date did you administer the last dose of the initial therapy?

Month	▼ January ...
Day	▼ January ...
Year	▼ January ...

Q19 Which of the following clinical signs did you see improvement in, or disappearance of, after completion of the initial therapy? (Please select all that apply)

- ☐ ⊗ I did not see improvement in any clinical signs
- ☐ Anisocoria (different sized-pupils)
- ☐ Blindness
- ☐ Color changes or spots in the eye
- ☐ Constipation/obstipation (difficulty defecating)
- ☐ Diarrhea
- ☐ Difficulty breathing
- ☐ Difficulty walking or jumping
- ☐ Distended abdomen (swollen belly due to fluid build-up)
- ☐ Hiding/lack of socialization
- ☐ Incontinence: fecal (loss of ability to control bowel movements)
- ☐ Incontinence: urinary (loss of bladder control)
- ☐ Increased water consumption
- ☐ Jaundice (yellowed skin or eyes)
- ☐ Lethargy (low energy)
- ☐ Loss of appetite
- ☐ Pale gums

- [ ] Paralysis (partial or total)
- [ ] Pica (eating/licking of inappropriate objects, eg. platic bags, paper, kitty litter)
- [ ] Seizures (either focal or grand mal)
- [ ] Tremors or shaking
- [ ] Upper respiratory issues (ie. sneezing, congestion, nasal discharge)
- [ ] Vomiting
- [ ] Weight loss
- [ ] Other: (please specify)

____

- [ ] ⊗No symptoms/issues were observed

Q20 Did your cat ever reach a clinical remission after the initial therapy (i.e., disappearance of all clinical signs)?

- ○ Yes
- ○ No

Q21 If yes, about how long was your cat in remission (free of clinical signs)?

- ○ Less than a week
- ○ Less than 2 weeks
- ○ Less than 4 weeks (one month)
- ○ Less than 2 months
- ○ Less than 6 months
- ○ Longer than 6 months, but less than a year
- ○ Longer than 1 year

---

*

Q64
What was your cat's weight at the beginning of the initial treatment?

Please specify weight in pounds ONLY. If you do not know your cat's weight, please enter "0".

Please provide weight in **decimals only**, NOT ounces (eg. 8.5 pounds instead of 8 pounds 8 ounces). **Only numeric responses with up to two decimals will be accepted.**

To convert weight from kilograms to pounds, multiply weight in kilogram by 2.2 (pounds (lbs.) = kilograms (kg.) x 2.2).

____

---

*

Q66
What was your cat's weight at the end of the initial treatment?

Please specify weight in pounds ONLY. If you do not know your cat's weight, please enter "0".

Please provide weight in **decimals only**, NOT ounces (eg. 8.5 pounds instead of 8 pounds 8 ounces). **Only numeric responses with up to two decimals will be accepted.**

To convert weight from kilograms to pounds, multiply weight in kilogram by 2.2 (pounds (lbs.) = kilograms (kg.) x 2.2).

____

End of Block: First Treatment of FIP

Start of Block: Second treatment

Q17 Did your cat receive a second round of therapy for FIP before starting molnupiravir therapy?

- ○ Yes
- ○ No

Q82 What was the second therapy used to treat your cat for FIP? (i.e. before starting molnupiravir)

- ○ Oral GS-441524
- ○ Injectable GS-441524
- ○ Combination of oral and injectable GS-441524
- ○ Combination of GS-441524 and GC
- ○ GC376
- ○ Remdesivir
- ○ Other (please specify) ____

Q83 What dose of GS-441524 therapy did you first use for your cat?

- ○ 4-5 mg/kg
- ○ 5-6 mg/kg
- ○ 6-7 mg/kg
- ○ 7-8 mg/kg
- ○ Other, please explain ____

Q84 How often did you administer GS-441524 therapy to your cat?

- ○ Once a day
- ○ Twice a day
- ○ Other, please explain ____

Q85 Did the dose of GS-441524 therapy change at any point during the course of treatment?

- ○ Yes
- ○ No

Q86 Please explain how the dosage of GS-441524 therapy changed during the course of treatment.

____

____

____

____

____

Q87 What dose and frequency of GC therapy did you use for your cat? Please indicate if this dose changed during the course of therapy.

____

Q88 What dose of GS-441524 and what dose of GC did you use to treat your cat?

- ◯ Dose of GS-441524 ____
- ◯ Dose of GC ____

Q89 How often did you administer GS-441524 and GC to your cat?

- ◯ Both therapies once a day
- ◯ Both therapies twice a day
- ◯ Other, please explain ____

Q90 What dose and frequency of remdesivir therapy did you use for your cat? Please indicate if this dose changed during the course of therapy.

____

Q92 What dose and frequency of this therapy did you use for your cat? Please indicate if this dose changed during the course of therapy.

____

JS

Q93 Second Therapy Section

What date did you start the second therapy for FIP? (ex: GS, GC, remdesivir)

Month	▼ January ...
Day	▼ January ...
Year	▼ January ...

Q94 Did your pet finish the standard 12 week (84 days) course of the second therapy?

◯ Yes

◯ No (Please explain) ____

Q95 Did you extend the course of the second therapy past 12 weeks (84 days)?

◯ Yes [Please include how long you extended treatment for (in days) below].
____

◯ No

Q96 Did you miss a dose of the second therapy for any reason?

◯ Yes (Please explain) ____

◯ No

JS

Q97 What date did you administer the last dose of the second therapy?

Month	▼ January ...
Day	▼ January ...
Year	▼ January ...

Q98 Which of the following clinical signs did you see improvement in, or disappearance of, after completion of the second therapy? (Please select all that apply)

- ☐ ⊗ I did not see improvement in any clinical signs
- ☐ Anisocoria (different sized-pupils)
- ☐ Blindness
- ☐ Color changes or spots in the eye
- ☐ Constipation/obstipation (difficulty defecating)
- ☐ Diarrhea
- ☐ Difficulty breathing
- ☐ Difficulty walking or jumping
- ☐ Distended abdomen (swollen belly due to fluid build-up)
- ☐ Hiding/lack of socialization
- ☐ Incontinence: fecal (loss of ability to control bowel movements)
- ☐ Incontinence: urinary (loss of bladder control)
- ☐ Increased water consumption
- ☐ Jaundice (yellowed skin or eyes)
- ☐ Lethargy (low energy)
- ☐ Loss of appetite
- ☐ Pale gums

- [ ] Paralysis (partial or total)
- [ ] Pica (eating/licking of inappropriate objects, eg. platic bags, paper, kitty litter)
- [ ] Seizures (either focal or grand mal)
- [ ] Tremors or shaking
- [ ] Upper respiratory issues (ie. sneezing, congestion, nasal discharge)
- [ ] Vomiting
- [ ] Weight loss
- [ ] Other: (please specify)

____

- [ ] ⊗No symptoms/issues were observed

Q99 Did your cat ever reach a clinical remission after the second therapy (i.e., disappearance of all clinical signs)?

- ○ Yes
- ○ No

Q100 If yes, about how long was your cat in remission (free of clinical signs)?

- ○ Less than a week
- ○ Less than 2 weeks
- ○ Less than 4 weeks (one month)
- ○ Less than 2 months
- ○ Less than 6 months
- ○ Longer than 6 months, but less than a year
- ○ Longer than 1 year

---

*

Q101
What was your cat's weight at the beginning of the second treatment?

Please specify weight in pounds ONLY. If you do not know your cat's weight, please enter "0".

Please provide weight in **decimals only**, NOT ounces (eg. 8.5 pounds instead of 8 pounds 8 ounces). **Only numeric responses with up to two decimals will be accepted.**

To convert weight from kilograms to pounds, multiply weight in kilogram by 2.2 (pounds (lbs.) = kilograms (kg.) x 2.2).

____

---

*

Q102
What was your cat's weight at the end of the second treatment?

Please specify weight in pounds ONLY. If you do not know your cat's weight, please enter "0".

Please provide weight in **decimals only**, NOT ounces (eg. 8.5 pounds instead of 8 pounds 8 ounces). **Only numeric responses with up to two decimals will be accepted.**

To convert weight from kilograms to pounds, multiply weight in kilogram by 2.2 (pounds (lbs.) = kilograms (kg.) x 2.2).

____

End of Block: Second treatment

Start of Block: Molnupiravir Treatment

*

Q68 Molnupiravir Therapy Protocol

What was your cat's weight at the beginning of molnupiravir treatment?

Please specify weight in pounds ONLY. If you do not know your cat's weight, please enter "0".

Please provide weight in **decimals only**, NOT ounces (eg. 8.5 pounds instead of 8 pounds 8 ounces). **Only numeric responses with up to two decimals will be accepted.**

To convert weight from kilograms to pounds, multiply weight in kilogram by 2.2 (pounds (lbs.) = kilograms (kg.) x 2.2).

____

Q74 Which of the following symptoms was your cat experiencing around the time molnupiravir therapy was started? (Please check all that apply)

- ☐ Anisocoria (different sized-pupils)
- ☐ Blindness
- ☐ Bloody stool
- ☐ Color changes or spots in the eye
- ☐ Constipation/obstipation (difficulty defacating)
- ☐ Cough
- ☐ Decreased appetite or refusal to eat
- ☐ Diarrhea
- ☐ Difficulty breathing
- ☐ Difficulty walking or jumping
- ☐ Distended abdomen (swollen belly due to fluid build-up)
- ☐ Hiding/lack of socialization
- ☐ Incontinence: fecal (loss of ability to control bowel movements)
- ☐ Incontinence: urinary (loss of bladder control)
- ☐ Increased water consumption
- ☐ Increase in urinations (volume and/or frequency)
- ☐ Jaundice (yellowed skin or eyes)

- [ ] Lethargy/listlessness (low energy)
- [ ] Loss of appetite
- [ ] Pale gums
- [ ] Paralysis (partial or total)
- [ ] Pica (eating/licking of inappropriate objects, eg. platic bags, paper, kitty litter)
- [ ] Seizures (either focal or generalized)
- [ ] Tremors or shaking
- [ ] Upper respiratory issues (ie. sneezing, congestion, nasal discharge)
- [ ] Vocalization
- [ ] Vomiting
- [ ] Weight loss
- [ ] Other: (please specify) ________________
- [ ] ⊗ No symptoms/issues were observed

JS

Q27 What date did you administer the first dose of molnupiravir?

Month	▼ January ...
Day	▼ January ...
Year	▼ January ...

Q70 What brand name of molupiravir was used for treatment?

- Aura 2801
- Aura 1931
- Rose EIDD
- Capella EIDD
- Other, please explain ____

Q18 What was the dose of molnupiravir therapy given to your cat at the beginning of treatment?

- ◯ Less than 6mg/kg (please list the dose in mg/kg) ____
- ◯ 6mg/kg
- ◯ 7mg/kg
- ◯ 8mg/kg
- ◯ 9mg/kg
- ◯ 10mg/kg
- ◯ 11mg/kg
- ◯ 12mg/kg
- ◯ 13mg/kg
- ◯ 14mg/kg
- ◯ 15mg/kg
- ◯ 16mg/kg
- ◯ 17mg/kg
- ◯ 18mg/kg
- ◯ 19mg/kg
- ◯ 20mg/kg
- ◯ Higher than 20mg/kg (please list the dose in mg/kg) ____

Q104 What was the dose of molnupiravir therapy given to your cat at the end of treatment?

○ Less than 6mg/kg (please list the dose in mg/kg)
____

○ 6mg/kg

○ 7mg/kg

○ 8mg/kg

○ 9mg/kg

○ 10mg/kg

○ 11mg/kg

○ 12mg/kg

○ 13mg/kg

○ 14mg/kg

○ 15mg/kg

○ 16mg/kg

○ 17mg/kg

○ 18mg/kg

○ 19mg/kg

○ 20mg/kg

○ Higher than 20mg/kg (please list the dose in mg/kg)
____

Q71 How often did you administer molnupiravir to your cat?

- ○ Once a day
- ○ Twice a day
- ○ Other, please explain ____

Q59 At any point during the course of therapy, did the dose or frequency of molnupiravir change?

- ○ Yes
- ○ No

Q101 Please briefly explain why the dose of molnupiravir changed over the course of therapy.

____

____

____

____

____

Q28 Did you finish the entire 8-10 week course of molnupiravir therapy?

- ○ Yes
- ○ No, my cat passed away or was euthanized
- ○ No, for another reason. (Please explain.) ____

Q102 Did you extend the course of therapy passed 8-10 weeks?

◯ Yes [Please include how long you extended treatment for (in days) below].

____

◯ No

Q29 Did you miss a dose of molnupiravir for any reason?

◯ Yes (Please explain) ____

◯ No

◯ Stopped treatment (death, euthanasia, or other reason)

JS

Q30 What date did you administer the last dose of molnupiravir?

Month	▼ January ...
Day	▼ January ...
Year	▼ January ...

Page Break

Q33 Molnupiravir Therapy Outcome

How soon did you notice an improvement in your cat's clinical signs after starting molnupiravir therapy?

- ◯ Less than 1 week
- ◯ Within 2 weeks
- ◯ Within 3 weeks
- ◯ Within 4 weeks
- ◯ Never saw improvement
- ◯ Other (please explain) ____

Q31 Which of the following clinical signs did you see improvement in, or disappearance of, after completion of molnupiravir therapy? (Please check all that apply)

- ☐ ⊗ I did not see improvement in any clinical signs
- ☐ Anisocoria (different sized-pupils)
- ☐ Blindness
- ☐ Bloody stool
- ☐ Color changes or spots in the eye
- ☐ Constipation/obstipation (difficulty defacating)
- ☐ Cough
- ☐ Decreased appetite or refusal to eat
- ☐ Diarrhea
- ☐ Difficulty breathing
- ☐ Difficulty walking or jumping
- ☐ Distended abdomen (swollen belly due to fluid build-up)
- ☐ Hiding/lack of socialization
- ☐ Incontinence: fecal (loss of ability to control bowel movements)
- ☐ Incontinence: urinary (loss of bladder control)
- ☐ Increased water consumption
- ☐ Increase in urinations (volume and/or frequency)

- [ ] Jaundice (yellowed skin or eyes)
- [ ] Lethargy/listlessness (low energy)
- [ ] Loss of appetite
- [ ] Pale gums
- [ ] Paralysis (partial or total)
- [ ] Pica (eating/licking of inappropriate objects, eg. platic bags, paper, kitty litter)
- [ ] Seizures (either focal or generalized)
- [ ] Tremors or shaking
- [ ] Upper respiratory issues (ie. sneezing, congestion, nasal discharge)
- [ ] Vocalization
- [ ] Vomiting
- [ ] Weight loss
- [ ] Other: (please specify)

- [ ] ⊗ No symptoms/issues were observed

Q54 How long did your cat's symptoms persist after completion of molnupiravir therapy?

- My cat's symptoms had resolved before the completion of molnupiravir therapy
- Less than 1 week after completion of molnupiravir therapy
- Less than 2 weeks after completion of molnupiravir therapy
- Less than 4 weeks (one month) after completion of molnupiravir therapy
- Less than 2 months after completion of molnupiravir therapy
- Less than 6 months after completion of molnupiravir therapy
- Longer than 6 months, but less than a year after completion of molnupiravir therapy
- Longer than 1 year after completion of molnupiravir therapy

Q48 After completion of molnupiravir therapy, which of the following of your cat's symptoms persisted? Please select all that apply.

- ☐ Anisocoria (different sized-pupils)
- ☐ Blindness
- ☐ Bloody stool
- ☐ Color changes or spots in the eye
- ☐ Constipation/obstipation (difficulty defacating)
- ☐ Cough
- ☐ Decreased appetite or refusal to eat
- ☐ Diarrhea
- ☐ Difficulty breathing
- ☐ Difficulty walking or jumping
- ☐ Distended abdomen (swollen belly due to fluid build-up)
- ☐ Hiding/lack of socialization
- ☐ Incontinence: fecal (loss of ability to control bowel movements)
- ☐ Incontinence: urinary (loss of bladder control)
- ☐ Increased water consumption
- ☐ Increase in urinations (volume and/or frequency)
- ☐ Jaundice (yellowed skin or eyes)

- [ ] Lethargy/listlessness (low energy)
- [ ] Loss of appetite
- [ ] Pale gums
- [ ] Paralysis (partial or total)
- [ ] Pica (eating/licking of inappropriate objects, eg. platic bags, paper, kitty litter)
- [ ] Seizures (either focal or generalized)
- [ ] Tremors or shaking
- [ ] Upper respiratory issues (ie. sneezing, congestion, nasal discharge)
- [ ] Vocalization
- [ ] Vomiting
- [ ] Weight loss
- [ ] Other: (please specify) ________________________
- [ ] ⊗No symptoms/issues were observed

Q34 Did your cat ever reach remission during/after molnupiravir therapy (i.e., disappearance of all clinical signs)?

○ Yes

○ No

Q71 Is your cat still in remission as of today?

- ○ Yes
- ○ No

Q35 If no, about how long was your cat in remission (free of clinical signs?)

- ○ Less than 1 week
- ○ Less than 2 weeks
- ○ Less than 4 weeks (one month)
- ○ Less than 2 months
- ○ Less than 6 months
- ○ Longer than 6 months, but less than a year
- ○ Longer than 1 year

*

Q69
What was your cat's weight at the end of molnupiravir treatment?

Please specify weight in pounds ONLY. If you do not know your cat's weight, please enter "0".

Please provide weight in **decimals only**, NOT ounces (eg. 8.5 pounds instead of 8 pounds 8 ounces). **Only numeric responses with up to two decimals will be accepted.**

To convert weight from kilograms to pounds, multiply weight in kilogram by 2.2 (pounds (lbs.) = kilograms (kg.) x 2.2).

____

Page Break

Q46 Wrap up on Molnupiravir Section

During the course of molnupiravir therapy, did your cat receive any of the following medications or treatments other than molnupiravir, including those administered directly by your veterinarian (eg. an antibiotic injection)? (Please check all that apply)

- ☐ Antibiotics (please specify type) ____
- ☐ Blood transfusion
- ☐ Anti-nausea medications (eg. cerenia, maropitant, ondansetron)
- ☐ Injectable steroids (eg. depomedrol, dexamethasone)
- ☐ Gabapentin/neurontin
- ☐ Fluid administration: subcutaneous (SC/SQ)
- ☐ Fluid administration: intravenous (IV)
- ☐ NSAID (eg. meloxicam, metacam, onsior)
- ☐ Oxygen therapy
- ☐ Polyprenyl Immunostiumland (PI) or VetImmune
- ☐ Oral steroids (eg. prednisolone): (please specify dose and frequency) ____
- ☐ T-cyte/Proboost/Thymic Protein A supplementation
- ☐ Vitamin B12 injections
- ☐ Other: (please specify) ____

☐ ⊗ No medications were received/administered

Q58 Did your veterinarian REPEAT any of these diagnostic tests while your cat was undergoing molnupiravir therapy? (Please select all that apply)

- ☐ Abdominal ultrasound
- ☐ Biopsy with IHC (immunohistochemical staining)
- ☐ Biopsy without IHC (immunohistochemical staining) - histopathology only
- ☐ CBC (complete blood count; a common type of blood test)
- ☐ Chemistry panel (AKA biochemical profile; a common type of blood test)
- ☐ CT (AKA "cat scan")
- ☐ Cytology (via fine needle aspirate)
- ☐ ECG/EKG (electrocardiogram)
- ☐ Feline coronavirus (FCoV) RT-PCR or titer
- ☐ FIP ELISA 7b protein test (by Antech Laboratories)
- ☐ FIP mRNA PCR or IFA (immunofluorscence assay)
- ☐ FIP Virus RealPCR test (by IDEXX Laboratories)
- ☐ Fluid analysis: cerebrospinal fluid (CSF) from a spinal tap
- ☐ Fluid analysis: complete fluid analysis of effusion fluid
- ☐ Fundic exam (a specific type of eye exam)
- ☐ Neurological exam
- ☐ MRI

☐ Radiographs (x-rays)

☐ Rivalta test (test conducted on effusion fluid)

☐ Other: (please specify)

____

☐ ⊗None - no specific follow-up tests were performed by my veterinarian

Q54 Do you have results from any of these diagnostic tests?

○ Yes (Please upload them at the end of the survey, if possible!)

○ No

Q32 At any point during or after molnupiravir treatment, was your cat euthanized or did they pass away?

○ Yes

○ No

Q55 Please describe how your cat is doing today?

____

____

____

____

____

Q49 If yes, please explain the circumstances of your cat's passing.

____

End of Block: Molnupiravir Treatment

Start of Block: Wrap Up

Q103 Final Thoughts

For what reason did you switch to treating your cat with molnupiravir therapy?

- ○ My cat failed/relapsed after trying a different therapy
- ○ My cat was not responding to treatment with a different therapy
- ○ My cat was not tolerating the injectable therapies
- ○ Molnupiravir was less expensive
- ○ Molnupiravir was easier to get ahold of
- ○ Other (please explain) ____

Q56 Did your cat experience any adverse effects of molnupiravir therapy?

- ○ Yes
- ○ No

Q57 What potential adverse effects did your cat experience with molnupiravir therapy? (Please select all that apply)

- ☐ Nausea and/or vomiting
- ☐ Diarrhea
- ☐ Lose of appetite
- ☐ Change in behavior
- ☐ Dizziness
- ☐ Other (please explain) ____

Q51 How satisfied are you with your experience of undergoing molnupiravir therapy for your cat?

- ○ Extremely dissatisfied
- ○ Somewhat dissatisfied
- ○ Neither satisfied nor dissatisfied
- ○ Somewhat satisfied
- ○ Extremely satisfied

Q53 What is your estimated cost (in U.S. dollars) for FIP treatment?

- ○ Estimated cost for initial medication (ex: GS-441524) ____
- ○ Estimated cost for 2nd round of medication (if not molnupiravir) ____
- ○ Estimated cost for molnupiravir therapy ____
- ○ Estimated cost on veterinary exams and diagnostics ____
- ○ Estimated cost on supportive care (fluids, vitamins, etc) ____

Q55 Please use this space to include any information you deem important and has not already been disclosed during this survey.

____

____

____

____

____

End of Block: Wrap Up

Start of Block: Uploading Diagnostics

Q50 Please upload any additional documentation that you are willing to share with us (ex: veterinary records, diagnostic results, treatment logs, etc) as they would be very helpful for our study. Your veterinarian's name and clinic will be kept strictly confidential, and they will not be contacted without your permission. No identifying information about you or your cat will be shared in any way.

Please upload any of your cat's diagnostic results or veterinary records here.

Q44 Please upload any of your cat's diagnostic results or veterinary records here.

Q9 Please upload any of your cat's diagnostic results or veterinary records here.

Q45 Please upload any of your cat's diagnostic results or veterinary records here.

Q46 Please upload any of your cat's diagnostic results or veterinary records here.

Q47 Please upload any of your cat's diagnostic results or veterinary records here.

Q48 Please upload any of your cat's diagnostic results or veterinary records here.

Q49 Please upload any of your cat's diagnostic results or veterinary records here.

End of Block: Uploading Diagnostics

Data S2: Cat #6 Abbreviated clinical history log

12.09.2021 started Lucky brand GS-441524 at 0.9ml

12.11.2021 we started 1.4mL instead of the initial 0.9mL. weight- 3 pounds

12.14.2021 we added 1/2 pill of molnupiravir every 12hrs, then cat #3 had 2 seizures that night and we discontinued molnupiravir and continued with 2.8mL GS-441524 twice a day

12.22.2021- we switched From Lucky (injectable GS-441524) to Aura 20 (injectable GS-441524). inject 2.8mL every 8 hrs weight- 3lbs 7 oz

12.24.2021- we increased Aura to 3mL every 12 hrs

01.06.2022-we increased Aura to 3.4mL every 12 hrs and added injectable GC "dark green top vial" 1.3mL twice a day weight- 3 lbs, 10 oz.

02.13.2022- we are still doing Aura 20, 2.5mL TWICE A DAY and started 4 GS-441524 Lucky brand 2kg pills every 6 hours and the GC "dark green top vial" 2mL

02.20.2022-increased GS-441524 pills to 5 pills 3 times a day weight- 5 pounds 7 oz

02.25.2022- discontinued GS-441524 pills because labs came back with values for renal failure and high phosphorus level.

03.05.2022- discontinued Aura 20 and used molnupiravir 1 pill twice a day with the GC dark green vial 2mL twice day

03.09.2022- discontinued GC dark green inject. just 1 pill of molnupiravir twice a day

03.30.2022- we have normal labs results

05.20.2022- we stopped all treatment and went into observation period

Data S3: Cat #21 Abbreviated clinical history log

Jan 19, 2022- she was switched to molnupiravir (aura 2801) at 13 mg/kg. She did well and improved dramatically for about 10 days

Jan 29, 2022 - Cat #21 showed signs of neurological relapse (loss of balance, rear leg weakness) dosage of Aura-2801 raised to 15 mg/kg

Jan 31, 2022 - continues to decline rapidly, 20 mg/kg SID GS-441524 and 5 mg pred added to therapy regimen. Signs of neuro pain seen.

Feb 3, 2022 - EIDD dosage raised to 18 mg/kg BID due to continued neuro decline

Feb 5, 2022 - EIDD discontinued. 20 mg/kg SID GS-441524 continued, 40 mg/kg BID GC added to therapy.

Feb 7, 2022 - slight improvement noted in walking, but still fecal incontinent

Feb 11, 2022 - GS-441524 dosage increased to 25 mg/kg SID, GC dosage increased to 55 mg/kg BID

Feb 15, 2022 - legs still unstable but bright, eating well, energetic

Feb 23, 2022 - begins jumping again

April 1, 2022 - pred discontinued

April 13, 2022 - Pica noted

April 18, 2022 - neurological pain noted. GS and GC discontinued, Aura EIDD-1931 started at 25 mg/kg BID

April 23/24, 2022 - large improvement in neuro symptoms, pain gone, energetic and running.

May 18, 2022 - heart murmur noted. Clinically large improvement in neuro symptoms. Will jump.

Jun 16, 2022 - itraconazole started for ringworm

Jun 24, 2022 - decreased appetite and possible change in gait, low energy dose increased on Aura EIDD-1931 to 30 mg/kg. Itraconazole stopped

July 1, 2022 - neurologically improved, willing to jump, energetic

July 23, 2022 - folded eartips noted. (likely EIDD side effect) EIDD therapy discontinued. Remaining clinical signs thought to be residual.

August 20, 2022 - disease free for 4 weeks before relapsing. Started on a second round of molnupiravir at the same dosage.
